# School Belongingness and Mental Health Functioning across the Primary-Secondary Transition in a Mainstream Sample: Multi-Group Cross-Lagged Analyses

**DOI:** 10.1371/journal.pone.0099576

**Published:** 2014-06-26

**Authors:** Sharmila Vaz, Marita Falkmer, Richard Parsons, Anne Elizabeth Passmore, Timothy Parkin, Torbjörn Falkmer

**Affiliations:** 1 School of Occupational Therapy and Social Work, Centre for Research into Disability and Society, Curtin Health Innovation Research Institute, Curtin University, Perth, Western Australia, Australia; 2 School of Occupational Therapy and Social Work, Curtin Health Innovation Research Institute, Curtin University, Perth, Western Australia, Australia; 3 School of Education and Communication, CHILD programme, Institution of Disability Research Jönköping University, Jönköping, Sweden; 4 School of Occupational Therapy and Social Work, and School of Pharmacy, Curtin Health Innovation Research Institute, Curtin University, Perth, Western Australia, Australia; 5 School of Occupational Therapy, La Trobe University, Melbourne, Vic., Australia; 6 Rehabilitation Medicine, Department of Medicine and Health Sciences (IMH), Faculty of Health Sciences, Linköping University and Pain and Rehabilitation Centre, UHL, County Council, Linköping, Sweden; Chiba University Center for Forensic Mental Health, Japan

## Abstract

The relationship between school belongingness and mental health functioning before and after the primary-secondary school transition has not been previously investigated in students with and without disabilities. This study used a prospective longitudinal design to test the bi-directional relationships between these constructs, by surveying 266 students with and without disabilities and their parents, 6-months before and after the transition to secondary school. Cross-lagged multi-group analyses found student perception of belongingness in the final year of primary school to contribute to change in their mental health functioning a year later. The beneficial longitudinal effects of school belongingness on subsequent mental health functioning were evident in all student subgroups; even after accounting for prior mental health scores and the cross-time stability in mental health functioning and school belongingness scores. Findings of the current study substantiate the role of school contextual influences on early adolescent mental health functioning. They highlight the importance for primary and secondary schools to assess students’ school belongingness and mental health functioning and transfer these records as part of the transition process, so that appropriate scaffolds are in place to support those in need. Longer term longitudinal studies are needed to increase the understanding of the temporal sequencing between school belongingness and mental health functioning of all mainstream students.

## Mental Health Problems in Adolescence

Worldwide estimates of mental health problems in children and youth range from 10–20% [1]. Australian figures report a 14% prevalence in a national sample of 4–12 year olds, which rises to 19% in the 13–17 year old category [2] and 27% in the 18–24 year old group [3]. These figures suggest that approximately one in four to five young Australians have a mental health problem [4]. Mental health functioning of children and youth has been shown to vary due to gender, presence of disability and household socio-economic standing (SES). For example, conduct disorder is the most common psychiatric disorder in childhood, with three times as many boys as girls being affected [5]. During adolescence, girls have a higher prevalence of depression and eating disorders, and engage more in suicidal ideation and suicide attempts than boys, who are more prone to engage in high risk behaviours and commit suicide more frequently [6], [7]. Young people with an intellectual disability manifest behaviours and experiences which may be indicative of mental health or psychological impairment three to four times more often than their typically developing peers; with psychiatric disorders in young people with a disability often undiagnosed and untreated [8]. Household-SES influences physical and mental health across the lifespan, with socially and economically disadvantaged children and adults found to be an increased risk for both physical and mental health problems [9]–[12]. Thus, it is imperative that research studies account for within group variability in mental health functioning of children and youth.

Of concern is the growing evidence on the stability of mental health problems in children and adolescents [13], [14] and its longitudinal effects on mental health disorders, delinquency, crime, unemployment, homelessness and suicidal behaviour in adulthood [14]–[22]. Mental health problems in children and adolescents could be antecedents of chronic, complex, disabling and expensive complications in adult life. For these reasons, early detection of clinical and subclinical mental health issues is important. Most mental health disorders that are likely to persist into adult life emerge between ages 12 and 25 [23], [24]. While early intervention is more economical and cost-effective than later action [25], its effectiveness in some cases is modest [26], [27] or fails to reach the majority of those most in need [26]. Australian data suggest that only one in four youth who need professional help actually get the help they need [28]. These facts, underscore the need to gain a deeper understanding of pathways in and out of childhood mental health problems [15], [28]–[30]. Schools are an ideal setting for efficiently detecting children and adolescents with unidentified mental health problems because they offer the opportunity to reach large numbers of students [2], [30], [31].

### School belongingness and overall mental health functioning across primary-secondary school transition

In recent years, school belongingness, referring to students’ beliefs of being “personally accepted, respected, included, and supported by others in the school social environment” [32], has emerged as an important factor associated with positive health outcomes [33], [34]. Cross-sectional studies document moderate associations between school belongingness and emotional distress and depression in typically developing adolescents [35]–[38], before and after accounting for personal and contextual factors, such as family-parent-belongingness, self-esteem and grade point average [36], [37]. Short term longitudinal studies present mixed findings on the directional relationships between these constructs. In some studies [39], [40] a unidirectional relationship has been documented; with school belongingness predicting selective prospective mental health components, depending on gender. For example, Shocket and colleagues [40] found that early adolescents’ perception of school belongingness predicted future depressive symptoms in boys and girls; anxiety in girls; and conduct problems in boys. Other studies suggest bidirectional relationships between these constructs, which vary depending on the type of mental health domain being measured [41]. Loukas and colleagues [41] presented evidence of a bidirectional loop between school belongingness and conduct problems, but not depressive symptoms in 10–14 year old typically developing youth [41]. Also, the positive effects of school belongingness have been found to extend to students’ home lives; concomitantly buffer the effects of family disadvantage on functioning [42]; and prospectively protect them from involvement in risk behaviours [43], [44]. Consequently, a growing body of evidence with typically developing youth supports the interrelationship between school belongingness and positive mental health outcomes.

Conspicuous in the above cited investigations on school belongingness and mental health functioning, is the exclusion of students with disabilities in the study samples, despite their presence in the regular school system for several decades. Additional research is needed to authenticate the role of school belongingness in the disability subgroup. Preliminary findings are hopeful, showing school belongingness to be negatively associated with emotional stress, suicide attempts, and violence amongst students with learning disabilities [45]. Yet another gap is the absence of evidence on the prospective benefits of fostering belongingness in primary school on overall mental health functioning of all students after the transition to secondary school, or whether there are student subgroups, based on gender, disability status, or household-SES, that need additional support.

Students in western societies, including Australia, negotiate the primary-secondary school transition at a time in development when they are striving to gain independence from their parents, establish their unique identity [46], [47], and gain approval and support from peers [48]. As a result of this school transition, students experience a disruption of the secure peer network forged in primary school and a remixing of friendship networks and social hierarchies. It is likely that students are forced to redefine their sense of school belongingness after they transition to secondary school. Whether poorer mental health functioning before the transition is associated with poorer school belongingness thereafter remains mainly unexplored.

## Aim and Objectives

The current study extends the existing knowledge base on primary-secondary school transition by explicitly examining the temporal relationships between school belongingness and overall mental health functioning after one year, by tracking a cohort with and without disability, enrolled in the regular school system in Western Australia (WA). We also tested the equivalence of these relationships across gender, disability and household-SES. It was hypothesised that:

- direct relationships would exist between concurrent perception of school belongingness and overall mental health functioning, before and after the transition; and
- primary school belongingness would be related to overall mental health functioning in early secondary school, even after accounting for prior mental health scores.

No hypothesis was made regarding the predictive role of mental health in primary school on school belongingness a year later, due to the inconsistent empirical evidence on this issue.

## Method

A cohort study using a prospective, longitudinal design with two data collection points was used [Primary school  =  Wave 1, and Secondary school  =  Wave 2]. Students enrolled in the final year of primary school in WA (class 6 or 7), in the academic years commencing January 2006 or 2007, and due to transition to either middle or secondary school in January 2007 or 2008, were considered for inclusion in the study. Inclusion was limited to regular schools in the educational districts of metropolitan Perth or other major city centres of Western Australia (WA). Several recruitment strategies were used to maximize reach and representativeness. The current study is part of a larger study on the factors associated with student academic, social-emotional and participatory adjustment across the primary-secondary school transition [49]. Details on the study design, recruitment and data collection have been published elsewhere [50]. For the ease of readership, a brief overview is described below.

Wave 1 data collection occurred six months prior to the transition to either middle or secondary school, with data collected from students (with and without disabilities) and a primary caregiver (parent or guardian). Wave 2 data were collected 6 months after the transition, using the same procedure and sample as Wave 1.

Information was collected via survey questionnaires, primarily paper and pencil format. Informed written consent was obtained from school principals, parents, teachers, and written assent was obtained from students to participate in this study. In situations where the student declined to participate, even with parental consent, they were not included. All participants were made aware that they were not obliged to participate, and were free to withdraw from the study at any time without justification or prejudice. Ethics approval was obtained from Curtin University Health Research Ethics Committee, in Western Australia (WA) (Reference number HR 194/2005).

At Wave 1, data were collected from 395 students from 75 primary schools across the Perth metropolitan area and major city centres across WA. An attrition rate of 32.7% resulted in a Wave 2 sample of 266 participants from 52 primary schools and 152 secondary schools. Chi-square and paired sample *t*-tests showed that the participants who continued to be involved in the study at Wave 2 did not differ from those who discontinued involvement, on gender, disability, household SES-level, school belongingness, and mental health functioning scores. The current study uses data from the 266 students that answered both Wave 1 and 2 questionnaires. Access to the complete dataset can be obtained by contacting the first author.

The mean age of students sample at Wave 1 was 11.89 years (SD = 0.45 years, median = 12 years), and that at Wave 2 was 12.9 years (SD = 0.57 years, median = 13 years). Boys constituted 46.6% (n = 124) of the sample; and 25.9% (n = 69) were reported by a parent or primary caregiver to have a disability. The predominant disabilities included asthma (18.8%), auditory disability (15.9%), Attention Deficit Hyperactivity Disorder/Attention Deficit Disorders (14.5%), learning disability (11.6%), Autism Spectrum Disorders (10.1%), and cerebral palsy (8.7%). The majority of the sample came from mid-range households, and reported a weekly income of $600–1,999 (58.3%, n = 154) [51]. Under one-third of the sample (33%, n = 87) came from high-SES households ($ 2000+/week) and 8.7%, n = 23 were from low-SES groupings ($ 1–599 per week).

The sample represented 52 different primary schools and 77 different classes distributed across metropolitan Perth and other city centres of WA. Based on the Commonwealth Department of Education, Employment, and Workplace Relations measure of relative socio-economic advantage and disadvantage [67], 21.4% (n = 57) of the sample came from schools located in the most affluent areas across Australia (10^th^ decide), 44% (n = 117) came from the 9^th^ decile; 17.7% (n = 47) were from the 7–8^th^ decide and 16.9% (n = 45) came from more disadvantaged areas (1–6^th^ decide). Forty-seven percent of the sample (n = 125) were enrolled in the public schools, 29% (n = 77) in Catholic schools, and the remaining 24% (n = 64) in independent/private schools. There was a movement out of government schools into Catholic and independent schools for secondary education; with 11.2% of students (n = 14) moving into Catholic schools and 28.8% (n = 36) moving into independent schools for their secondary education.

### Data collection instruments

#### Mental health functioning

The 25-item parent version of the Strengths and Difficulties Questionnaire (SDQ) was used to measure student overall functioning across hyperactivity, emotional health, conduct problem and peer problem domains [52]. This version has moderate to high internal consistency scores (α = .70–80) [53], and is reported to be more sensitive than the Child Behaviour Check List [54] in detecting inattention and hyperactivity, and equally effective in detecting internalising and externalising problems in children and adolescents [55]. Established reliability and validity of the SDQ makes it a useful brief screening measure of adjustment and psychopathology in children and adolescents [53], [55]–[57]. Higher SDQ scores indicate poorer mental health functioning.

#### School belongingness

The 18-item, Psychological Sense of School Membership scale (PSSM) was used to assess students’ perceptions of belongingness in school [32]. The PSSM has satisfactory internal consistency (α = .803) [32]. Test-retest reliability indices of .78 (4-week interval) [58], and .56 and .60 for boys and girls respectively (12-month interval) have been documented in early adolescent samples [40]. The total PSSM scores correlate positively with school success [32], [59], lower levels of depression [40], and lower levels of anxiety [34]. Higher PSSM scores indicate better perceived school belongingness.

#### Family demographics and school contextual characteristics

Items were drawn from the Indicators of Social and Family Functioning Instrument Version-1 (ISAFF) [60] and Australian Bureau of Statistics (ABS, 2001) surveys, and used to provide family demographic information. Parents reported details on the family demographic characteristics, residence post code, and their child’s disability. Information on the school sector, post code number of students enrolled in each school, and organisational structure at each school was obtained from Department of Education and Training, WA records. The sample was categorised into three-income groups as per the median income distribution based on the Australian Bureau of Statistics [51] data.

### Data Management

Data were managed and analysed using the SPSS Version 20.0 and SAS Version 9.2 software packages. Only 1.8–2.5% of data were missing at scale levels. The estimation maximization algorithm and Little’s chi-square statistic revealed that the data were missing completely at random [61], [62]. Missing data replacement was undertaken using guidelines recommended by the SDQ tool developers (http://www.sdqinfo.org/c1.html). In the case of the school belongingness questionnaire, individual mean score substitution was used [63]. The validity of the data substitution techniques used was substantiated using sensitivity analyses.

In the present study, the bidirectional associations between school belongingness and mental health functioning over one year were estimated by cross-lagged analyses, using the structural equation modelling program, AMOS 5.0. A critical preliminary step in the analysis was to investigate if data met the normality assumption. With regard to the normality assumptions of the Full Information Maximum Likelihood estimation procedure, the normality of each variable was investigated in terms of its kurtosis and skewness [64]. Box-cox transformations were undertaken to normalise the PSSM and SDQ scores. In order to provide clinically relevant information, standardized Beta values from multiple linear regression analyses have also been presented, using the original data.

## Statistical Analyses

### Characteristics of the sample

Descriptive statistics were used to summarise the profile of participants.

### Testing for the effects of nesting of students on mental health and school belongingness

In order to test for the effect of clustering of students, i.e., nesting of students in classes within schools on their school belongingness (PSSM) and mental health functioning (SDQ) scores, a Hierarchical Linear Model was fitted using the mixed procedure in SAS. The class-level Intra Class Correlation Coefficients (ICC) for PSSM and SDQ were obtained, after adjustment for gender, disability, and household-SES.

Interrelationship between school belongingness (PSSM) and mental health functioning scores (SDQ). Pearson correlation coefficients were used to identify associations between the SDQ and PSSM scores at and between each wave. A two-factor analysis of variance with and without interaction terms was run to test the within-group variability in SDQ due to gender, disability, and household-SES.

**Figure 1:**
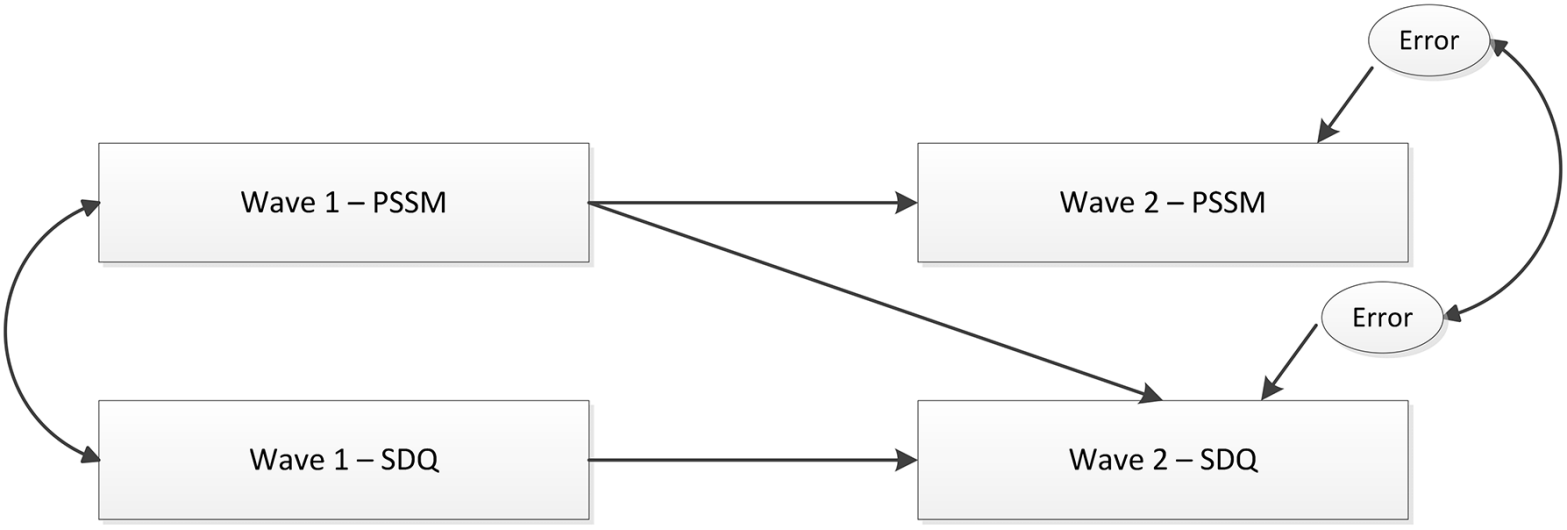
Cross-lagged relationship between PSSM and SDQ across the primary-secondary school transition.

Testing the hypothesized model of the relationship between school belongingness and mental health functioning. Autoregressive cross-lagged panel analysis was performed to study the reciprocal relationship between school belongingness and mental health functioning across the primary-secondary school transition. The path-diagram of the autoregressive cross-lagged model used in this study is presented in Figure 1.

Cross-lagged panel analysis allows examination of the cross-lagged paths while controlling for cross-time stability of each of the variables. In each of the models, the exogenous variables of Wave 1, which included school belongingness (PSSM) and mental health functioning (SDQ), were freely correlated. The residuals (error variances) of all Wave 2 variables were also correlated, due to auto-correlation effects. Stability paths from each of the Wave 1 constructs to their respective Wave 2 outcomes were included to partial out the effects of baseline adjustment problems. The inclusion of stability paths provides a stringent test of the Wave 1 influences and results in the examination of change in the variable of interest. To test the contribution of prior school belongingness (PSSM) to future mental health functioning (SDQ), paths from Wave 1-PSSM to Wave 2-SDQ were included. The opposite direction of associations, path from Wave 1-SDQ to Wave 2-PSSM was also simultaneously estimated, but not presented in Figure 1.

#### Multi-group invarian*c*e (equivalence) of the baseline model (Model 1)

To examine the equivalence of the hypothesized model across subgroups, namely, gender, disability and household-SES, parameters were simultaneously estimated for each subgroup, respectively. The fit of this simultaneously estimated unconstrained model provides the baseline value for each subgroup against which all subsequently specified models are compared. A fully constrained model, in which all parameters (factor variances, factor covariances, and error covariances) were constrained or specified to be equivalent across subgroups, was then calculated. χ^2^ difference tests were used to determine significant differences between the unconstrained and constrained models of each subgroup.

#### Model Evaluation Criteria

To determine the fit of the models, criteria were adopted from several sources. Because χ^2^ is influenced by sample size, we examined the χ^2^/degrees of freedom (df) ratio (χ^2^/df) rather than the significance of the χ^2^ alone [65]. Furthermore, we also used the Non-Normed Fit Index (NNFI) [66]. Additionally, fit was evaluated by one absolute fit index (the Root Mean Square Error of Approximation, RMSEA) and one incremental fit index (the Comparative Fit Index, CFI). An absolute fit index assesses how well a model reproduces the sample data without comparison to a reference model whereas an incremental fit index compares the target model to a more restricted baseline model [67]. Both these indexes take into account *model complexity*, which is an important property for comparing several alternative models with different degrees of complexity. According to criteria outlined by Hu and Bentler [67], a good fitting model has NNFI values of .95 or greater, RMSEA values smaller than .06, and a CFI greater than or equal to .95. In reporting on evidence of invariance, two criteria were used. Firstly, the multi-group model must exhibit an adequate fit to the data. Secondly, the determination of multi-group invariance is based on *delta* CFI; that is, when the differences in CFI values between models are less than .01 [68].

## Results

### Testing for the effects of nesting of students on mental health and school belongingness

A total of 52 different schools, and 77 different classes were involved in Wave 1. In order to test for the effect of clustering of students, i.e., nesting of students in classes within schools, a Hierarchical Linear Model was fitted using the mixed procedure in SAS. The class-level Intra Class Correlation Coefficients (ICC) for school belongingness and mental health functioning scores were obtained (after adjustment for the demographic data: gender, disability, and household-SES). The ICC for each model was low, ranging from 0–12%, showing that the contribution of the clustering to the overall variance was small, and therefore the clustering appeared to have minimal effect on the relationships between the student-level variables and school belongingness and mental health functioning scores. Hence, further analyses were undertaken at the level of the individual student.

### Characteristics of the sample: Within-group variability in mental health functioning

The mental health functioning scores (SDQ) of the students involved in the current study was better than those found in an Australian community sample for this age range [53], [69]. Within-group variability interactions were not statistically significant; hence only the main effects were included in the final models. In the case of Wave 2-SDQ scores, significant differences due to gender, *F* (1,256) = 4.30, *p* = 0.04, disability, *F* (1,256) = 49.95, *p* = <.001, and household-SES, *F* (2,254) = 3.77, *p* = 0.02 were found. Boys (*M = *8.88, *SE = *.45) had worse Wave2-SDQ scores than girls (*M = *7.65, *SE = *.44); and students with disability had worse scores (*M = *10.61, *SE = *.58) than those without disability (*M = *5.91, *SE = *.35). Students from low-SES households (*M = *8.90, SE = 1.18) had significantly poorer Wave2-SDQ scores than their peers from high-SES (*M* = 7.12, *SE* = .55, *p* = .05), but not mid-SES households (*M = *7.52, *SE* = .420, *p*>.05).

### Interrelationship between school belongingness (PSSM) and mental health functioning scores (SDQ)

The means, standard deviations and correlation matrix for all study variables without adjustment for gender, disability and household-SES are presented in Table 1. School belongingness (PSSM) was concurrently and longitudinally associated with mental health functioning (SDQ) at both waves of the study. Early adolescents reporting higher levels of school belongingness (higher PSSM) also reported better mental health functioning (lower SDQ). Examination of the cross-time stability of the variables indicated that the magnitude of the correlations was moderate for students’ perceptions of school belongingness (PSSM, *r = .*49), and larger for mental health functioning (SDQ, *r = .*77).

**Table 1:** Means, Standard Deviations, and correlations between the Strength and Difficulties Questionnaire (SDQ) and Psychological Scale of School Membership (PSSM) at Wave 1 and Wave 2.

	*M*	*SD*	*Wave 1 -SDQ*	*Wave 1 -PSSM*	*Wave 2-SDQ*	*Wave 2-PSSM*
*Wave1-SDQ*	6.90	5.56	1	−.42**	.77**	−.28**
*Wave1-PSSM*	3.90	.70		1	−.40**	.49**
*Wave2-SDQ*	7.11	5.24			1	−.33**
*Wave2-PSSM*	3.84	.64				1

**Correlation is significant at the 0.01 level (2-tailed).

*Note that higher SDQ indicate worse mental health functioning; higher PSSM indicate better school belongingness.*

### Testing the hypothesized model of the relationship between school belongingness and mental health functioning (Figure 1)

**Figure 2:**
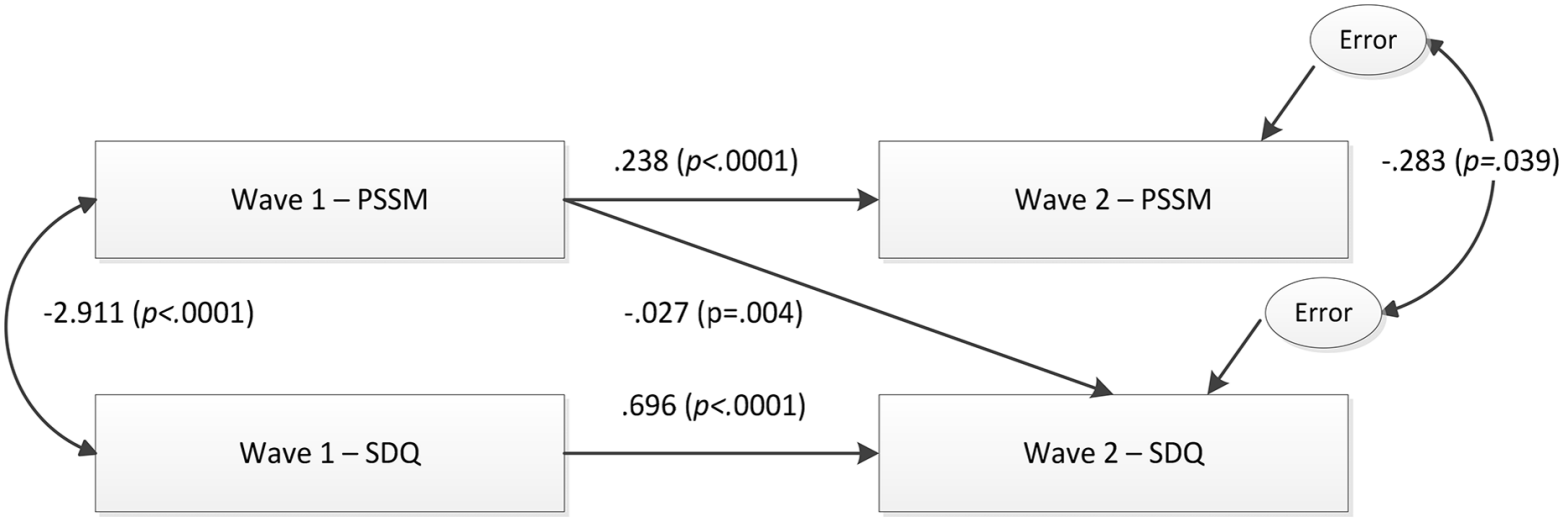
Cross-lagged relationship between PSSM and SDQ across the primary-secondary school transition, using data from the entire sample.

Figure 2 presents the most parsimonious baseline model that best fitted the data [χ^2^ (1, n = 266)  = .716, n.s.; CFI = 1.00; RMSEA = .000; AIC = 26.716]. As shown in Fig. 2, the stability paths were positive and significant, and inter-correlations among the two exogenous variables were significant. The error variance between the endogenous variables was significant and in the expected direction. Regarding the cross-lagged paths, Wave 1-PSSM was associated with lower levels of Wave 1-SDQ, even after controlling for baseline levels of all variables and for their cross-time stability. Clinically, this means that even after accounting for past mental health functioning (SDQ), a unit increase of Wave 1 school belongingness (PSSM) is associated with a corresponding 0.11 standard unit deviation (Beta) reduction in Wave 2-SDQ (based on multiple regression analyses). These results suggest that promoting school belongingness before the transition to secondary school has a beneficial effect on post-transition mental health functioning. The pathway from Wave 1-mental health (SDQ) to Wave 2-belongingness (PSSM) was not significant, as expected.

### Step 2: Multi-group invariance (equivalence) of the baseline model (Model 1)

Several additional models were examined to determine the equivalence of Figure 2 across gender (Table 2), disability (Table 3), and household-SES (Table 4). The fit of the unconstrained model in each analysis was compared to the fit of a fully constrained model. Imposing the equality constraints did not significantly deteriorate the fit of the model. Both models represented an excellent fit to the data. χ^2^ difference tests found no significant differences between the unconstrained and constrained models of each subgroup, suggesting invariance of the baseline model (Figure 2) across gender, disability and household-SES.

**Table 2:** Multi-group analyses of the unconstrained and constrained longitudinal relationship between school belongingness and mental health functioning across the primary-secondary school transition (Gender as the grouping variable).

Model no	Name of model	χ^2^	df	p	NFI	CFI	RMSEA	AIC
1	Unconstrained boys/girls	1.709	3	.635	.997	1.000	.000	55.709
2	Constrained	10.787	21	.967	.983	1.000	.000	28.787
3	Difference between 1 and 2	9.078	18	.957		0.000		

**Table 3:** Multi-group analyses of the unconstrained and constrained longitudinal relationship between school belongingness and mental health functioning across the primary-secondary school transition (Disability as the grouping variable).

Model no	Name of model	χ^2^	df	p	NFI	CFI	RMSEA	AIC
1	Unconstrained disability/no disability	1.877	3	.598	.997	1.000	.000	55.877
2	Constrained	16.35	21	.750	.974	1.000	.000	34.350
3	Difference between 1 and 2	14.477	18	.697		0.000		

**Table 4:** Multi-group analyses of the unconstrained and constrained longitudinal relationship between school belongingness and mental health functioning across the primary-secondary school transition (Household-SES as the grouping variable).

Model no	Name of model	χ^2^	df	p	NFI	CFI	RMSEA	AIC
1	Unconstrained for each SES group	10.877	27	.621	.983	1.000	.000	64.877
2	Constrained	31.266	9	.453	.952	1.000	.004	49.266
3	Difference between 1 and 2	20.389	18	.311		0.000		

## Discussion

The present study extends existing research by providing evidence that students’ ratings of belongingness in the final year of primary school contributes to change in their mental health functioning a year later. The beneficial effect of primary school belongingness on subsequent mental health functioning was evident for the entire population of mainstream students, even after accounting for their prior mental health scores and the cross-time stability in mental health functioning and school belongingness scores.

Findings of the current study corroborate a large body of evidence on the significance of boosting school belongingness as a mental health promotion strategy not only in typically developing students [36], [39], [40], [70], [71], but also students with disabilities. These results are of significance given current estimates that psychiatric disorders in young people with disabilities are often undiagnosed and untreated, despite the fact that these students manifest behaviours and experiences indicative of mental illness or psychological impairment three to four times more often than their typically developing peers [8]. Several theoretical underpinnings may explain the results. Students who sense a bonding in school are more likely to forge supportive relationships with teachers [32], [72], associate with pro-social peer groups [73] and are more likely to have better mental health functioning [74]. Students with social attachment to the school could be expected to feel committed to its goals, norms, and morals [75]–[77]. Hence, they are more likely to be involved in activities that enhance school belongingness. For this reason, they show fewer mental health problems than their counterparts who are not participating to the same extent. The positive effect of school belongingness on mental health may also represent the degree to which schools are meeting the developmental needs of their students [78], [79]. The associations between school belongingness and subsequent mental health functioning found in the current study suggest that both primary and secondary schools have a responsibility to foster school belongingness of all students from an early age, to safeguard future mental health.

Our results are consistent with the work of Shochet and colleagues [40] who reported significant relationships between prior school belongingness and future mental health symptoms in a large community sample (*N* = 2,200) of 12–14 year old Australian high school students. Shocket et al., [40] however used hierarchical linear modeling to test the relationship between the study variables, independently for boys and girls. The current study extends Shocket and colleagues work [40] in two ways. Firstly, it explicitly tested the role of gender, disability and household-SES as a moderator of the associations between school belongingness and early adolescent mental health functioning. Secondly, it applied multi-group cross-lagged panel analysis and took into account the commonly reported co-variation between school belongingness and mental health, at all points in time, along with the cross-lagged and cross-time stability of the variables. In doing so, confidence that the obtained associations reflect the unique contributions of the relationship between the study variables increases.

The current study’s findings are however contrary to those reported by Loukas et al., [41], who in a US sample of 9–14 year old youth, reported bidirectional relationships between school belongingness and conduct problems. One possible explanation for the absence of the significant cross-lagged association between prior mental health functioning and future school belongingness in the current study could be the highly stable level of mental health functioning across time. Alternative possible explanation for the null finding may be that other variables not examined in this study, such as grade point average, motivational variables, teachers’ classroom management strategy, etc. are better predictors of change in early adolescents’ school belongingness [80], [81]. Albeit, the high co-variations between Wave 1 measures could suggest that baseline reports of school belongingness contribute to initial levels of mental health functioning, which then remain stable across time. This could mean that failure to connect to the school during the early school years year may contribute to concurrent levels of mental health functioning, which are maintained across time. However, a parallel possibility is that initial of mental health functioning may influence initial perceptions of school belongingness, which are then maintained across time and re-enforce future mental health, cannot be ignored. For example, earlier studies have shown students with externalizing mental health problems to be more likely to experience peer rejection [82], higher levels of student–teacher conflict, and decreased levels of closeness [83]. The two-point study design precluded studying the longitudinal relationship between these constructs. Longer term time series analyses are desirable to parcel out these contributions and identify time-snaps that are ideal to intervene.

The high stability of students’ mental health functioning over time, noticed in our study and past research, highlights the need for primary and secondary schools to transfer students’ mental health functioning details as part of the transition transfer process, and factor them into their Individual Education Plans. The 12-month school belongingness stability correlation was slightly lower than previously documented in Australian community samples [40]. This could be attributed to the school sector change noted in the current study, i.e., shift from government to independent schooling for secondary education. Nonetheless, unlike previous studies that report significant declines in mean school belongingness scores as students’ progress through the secondary years of school [43], [84], [85], there was no significant between-group change, or within-subject change in school belongingness scores across the transition in the current study (Please contact first author for detailed results). Taken together, these findings highlight the need for schools to assess all students’ perceptions of school belongingness and mental health functioning before the transition, and ensure that student records are transferred as part of their Individualised Education Plan, so that appropriate scaffolds are in place to support those in need.

## Limitations

Detailed limitations of the current study have been discussed in an earlier publication [50]. Key points are hereafter discussed. For example, the current study’s population was drawn from metropolitan and other major city centres across WA, and did not involve other rural and regional populations, or major metropolitan cities in Australia; thus limiting generalizability. Despite several recruitment efforts, 70% of the schools declined to participate in the study, which may have introduced a possible bias. The study’s cohort was different to the profile of all schools in WA. The number of students in the lower SES subgroup was relatively small for sub-group differences to be identified. Furthermore, the criterion for inclusion into the disability category (i.e., limiting inclusion to those with a medical diagnosis who attended regular school for 80% of school hours) could have resulted in the exclusion of students with more disability related physical, cognitive, social, and emotional restrictions [86]. Furthermore, parents were asked to report on their child’s disability and overall mental functioning. Additional studies that involves multisource data from students, parents, teachers, and possibly validation using clinical interviews, medical and school records, are warranted to validate the findings [87]. The two-point longitudinal study design did not permit the study of the longer-term effect of transition on school belongingness and mental health functioning. Future research into the relationship between covariates (gender, disability and household-SES) in the cross-lagged model is desirable. The small size of the sub-group samples, together with the absence of any significant interactions between the covariates, precluded the need for those analyses in the present study. Longer term longitudinal studies that track students along the educational continuum are desirable to increase our understanding of how and when risk is expressed as disorder; to determine the ideal time to intervene; and the relevance of intervention on student outcomes.

## Conclusions

The current study adds to the growing body of research examining the role of school contextual influences in early adolescent mental health functioning. Adolescent experiences of belonging to, and closeness with, others at the school may buffer or offset the subsequent negative mental health functioning, above and beyond prior mental health functioning. The current study’s findings highlight the importance for both primary and secondary schools to assess the belongingness and mental health needs of all their students. Such assessments could allow schools to pay special attention to those with poorer mental health functioning and school belongingness scores, as these are more likely to continue to be disadvantaged over time.
